# 16461 Comparison of voxel intensity standardization methods in head and neck cancer magnetic resonance imaging

**DOI:** 10.1017/cts.2021.516

**Published:** 2021-03-30

**Authors:** Kareem A. Wahid, Renjie He, Brigid A. McDonald, Brian M. Anderson, Travis Salzillo, Sam Mulder, Jarey Wang, Christina Setareh Sharafi, Lance A. McCoy, Mohamed A. Naser, Sara Ahmed, Keith L. Sanders, Abdallah S.R. Mohamed, Yao Ding, Jihong Wang, Kate Hutcheson, Stephen Y. Lai, Clifton D. Fuller, Lisanne V. van Dijk

**Affiliations:** The University of Texas MD Anderson Cancer Center

## Abstract

ABSTRACT IMPACT: This work will standardize necessary image pre-processing for diagnostic and prognostic clinical workflows dependent on quantitative analysis of conventional magnetic resonance imaging. OBJECTIVES/GOALS: Conventional magnetic resonance imaging (MRI) poses challenges for quantitative analysis due to a lack of uniform inter-scanner voxel intensity values. Head and neck cancer (HNC) applications in particular have not been well investigated. This project aims to systematically evaluate voxel intensity standardization (VIS) methods for HNC MRI. METHODS/STUDY POPULATION: We utilize two separate cohorts of HNC patients, where T2-weighted (T2-w) MRI sequences were acquired before beginning radiotherapy for five patients in each cohort. The first cohort corresponds to patients with images taken at various institutions with a variety of non-uniform acquisition scanners and parameters. The second cohort corresponds to patients from a prospective clinical trial with uniformity in both scanner and acquisition parameters. Regions of interest from a variety of healthy tissues assumed to have minimal interpatient variation were manually contoured for each image and used to compare differences between a variety of VIS methods for each cohort. Towards this end, we implement a new metric for cohort intensity distributional overlap to compare region of interest similarity in a given cohort. RESULTS/ANTICIPATED RESULTS: Using a simple and interpretable metric, we have systematically investigated the effects of various commonly implementable VIS methods on T2-w sequences for two independent cohorts of HNC patients based on region of interest intensity similarity. We demonstrate VIS has a substantial effect on T2-w images where non-uniform acquisition parameters and scanners are utilized. Oppositely, it has a modest to minimal impact on T2-w images generated from the same scanner with the same acquisition parameters. Moreover, with a few notable exceptions, there does not seem to be a clear advantage or disadvantage to using one VIS method over another for T2-w images with non-uniform acquisition parameters. DISCUSSION/SIGNIFICANCE OF FINDINGS: Our results inform which VIS methods should be favored in HNC MRI and may indicate VIS is not a critical factor to consider in circumstances where similar acquisition parameters can be utilized. Moreover, our results can help guide downstream quantitative imaging tasks that may one day be implemented in clinical workflows.

